# Simulating stochastic adsorption of diluted solute molecules at interfaces

**DOI:** 10.1063/5.0064140

**Published:** 2022-01-11

**Authors:** Jixin Chen

**Affiliations:** Department of Chemistry and Biochemistry, Nanoscale and Quantum Phenomena Institute, Ohio University, Athens, Ohio 45701, USA

## Abstract

This report uses Monte Carlo simulations to connect stochastic single-molecule and ensemble surface adsorption of molecules from dilute solutions. Monte Carlo simulations often use a fundamental time resolution to simulate each discrete step for each molecule. The adsorption rate obtained from such a simulation surprisingly contains an error compared to the results obtained from the traditional method. Simulating adsorption kinetics is interesting in many processes, such as mass transportation within cells, the kinetics of drug–receptor interactions, membrane filtration, and other general reaction kinetics in diluted solutions. Thus, it is important to understand the origin of the disagreement and find a way to correct the results. This report reviews the traditional model, explains the single-molecule simulations, and introduces a method to correct the results of adsorption rate. For example, one can bin finer time steps into time steps of interest to simulate the fractal diffusion or simply introduce a correction factor for the simulations. Then two model systems, self-assembled monolayer (SAM) and biosensing on the patterned surface, are simulated to check the accuracy of the equations. It is found that the adsorption rate of SAM is highly dependent on the conditions and the uncertainty is large. However, the biosensing system is relatively accurate. This is because the concentration gradient near the interface varies significantly with reaction conditions for SAMs while relatively stable for the biosensing system.

## INTRODUCTION

**FIG. 1.**
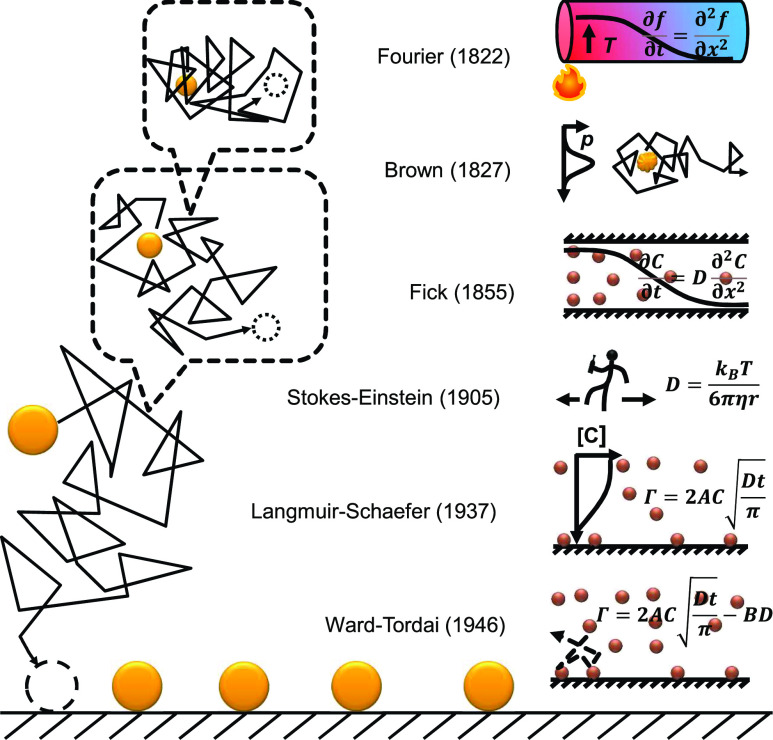
A brief early history of the random walk theory of diffusion and adsorption. The left shows the fractal nature (self-similarity over time) of diffusion and the right shows selected early events.

Molecules in diluted solution are moving constantly and randomly (Fig. 1). When still, a major contributor of this motion, diffusion, is fundamentally relevant in many fields such as cell biology, biosensing, separation, fluidic dynamics, reaction kinetics, catalysis, and batteries.^1–9^ The ensemble kinetics of diffusion has been summarized by Fick’s laws of diffusion.^2,3^ For example, the diffusion of materials in a diluted solution from a high-concentration reservoir into a tubing space forms a time- and space-dependent concentration gradient function *C*(*x*, *t*)^2,3^ derived from the heat equation formulated by Joseph Fourier in 1822,^3^

$$\frac{\partial C\left(x,t\right)}{\partial t}=\frac{\partial }{\partial x}\left[D\left(x,t\right)\frac{\partial C\left(x,t\right)}{\partial x}\right],$$ (1)

where *t* is time (unit s), *x* is the distance away from the interface (m), *C*(*x*, *t*) is the concentration gradient (molecules m^−3^), and *D*(*x*, *t*) is the time- and-space-dependent diffusion coefficient (m^2^ s^−1^), a constant under many conditions. Note that these units are for the 1D simplification of a 3D diffusion when the diffusions of the other two dimensions do not matter. For a real 1D diffusion, *C* will have the unit molecules m^−1^.

The special case with constant *D* has a formal analytical solution,^2^

$$C\left(x,t\right)=\frac{1}{\sqrt{4\pi Dt}}e^{-\frac{x^{2}}{4Dt}},$$ (2)

where *C*(*x*, *t*) (unit mol/m^3^) is the concentration of the molecules at space *x* (m) from origin *x* = 0 and time *t* from initial time *t* = 0 when all molecules are at the origin. Equation (2) is a 1D Gaussian distribution function with the standard deviation $\sigma =\sqrt{2Dt}$ and is normalized to the unit in all *x* spaces, i.e., at any giving snapshot of *t*, $A\int_{-\infty }^{\infty }C\left(x,t\right)dx=1$ (mol), where *A* = 1 is a unit area (m^2^).

Statistically, connecting the ensemble diffusion with single-molecule diffusion probability function using the ergodic principle, this concentration profile represents the probability density function (PDF) of a single molecule diffusing from the origin into space over time. It is a very important achievement in history that Stokes and Einstein come up with a single-particle random walking model to predict the diffusion constant of particles doing Brownian motion.^10^ The diffusion constant *D* for molecules, colloids, proteins, or much bigger particles in a solution can be estimated by the Stokes–Einstein equation,^11,12^

$$D=\frac{k_{B}T}{6\pi \eta r},$$ (3)

where *k*_B_ is the Boltzmann constant, *T* is temperature, *η* is the viscosity of the solution, and *r* is the radius of the particle. For a molecule approximated to a small ball, *r* (m) can be estimated from the molecular weight $Mw=\frac{4}{3}\pi r^{3}\rho$ (kg), where *ρ* (kg/m^3^) is the density of the neat molecule in the solid or liquid state (all in SI units).

An interesting application of this diffusion theory is to predict the adsorption rate of molecules in a diluted solution to a solid surface. In 1937, Langmuir and Schaefer came up with an equation to predict the adsorption rate at the short-time limit (a continuous model).^13^ Langmuir and Schaefer obtained adsorption kinetics by directly integrating Fick’s second law equation at a surface, assuming to absorb any molecules that have “crossed” it.^13,14^ The time-dependent concentration gradient at the surface is

$${\left(\frac{\partial c}{\partial t}\right)}_{s}=\frac{-DAc_{b}}{\sqrt{\pi Dt}}.$$ (4)

Integrating this equation over time, Langmuir and Schaefer gave the following equation:^13^

$$\Gamma \left(t\right)=2Ac_{b}\sqrt{\frac{Dt}{\pi }},$$ (5)

where *Γ*(*t*) (unit mol) is the number of molecules adsorbed on an area of surface *A* (unit m^2^) at time *t* (s), *c*_b_ (mol m^−3^) is the concentration of the adsorbate in the bulk solution, and *D* is the diffusion constant (m^2^ s^−1^).

In 1946, Ward and Tordai added a back-diffusion term in the equation to account for the adsorption during the longer period (Fig. 1),^14^

$$\Gamma \left(t\right)=2Ac_{b}\sqrt{\frac{Dt}{\pi }}-A\sqrt{\frac{D}{\pi }}\int_{0}^{\sqrt{t}}\frac{c\left(\tau \right)}{\sqrt{t-\tau }}d\tau ,$$ (6)

where *c*(*τ*) is the sub-surface molar concentration of the adsorbate near the surface (mol m^−3^) and *τ* is a dummy variable with the unit of time (s).

**FIG. 2.**
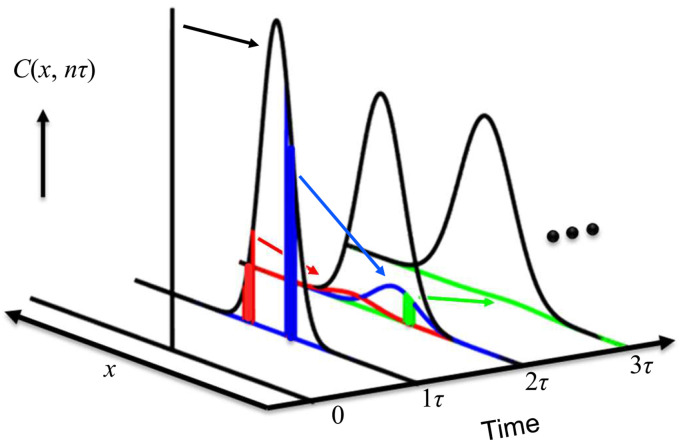
Self-similar fractal nature of the ensemble 1D diffusion expressed with the Crank–Nicolson method. Localized molecules at time 0 diffuse into a Gaussian concentration profile at desecrated time *τ* over space *x*. Subgroups of these molecules (two examples are colored red and blue) then further diffuse into the profiles at time 2*τ*, where combining all molecules gives the black probability profile [Eq. (2)]. At time 2*τ*, a subgroup of the blue molecules is labeled in green, which further diffuses into the green profile at time 3*τ*. This process continues until time *t* of interest. If *τ* is further divided into a smaller fraction of time *τ*′, a self-similar process evolves between the discrete times of *nτ* and *nτ*′.

Because our current measuring techniques, especially various chemical imaging methods, have discrete integration times,^4,6,9,15–24^ finding the correlation between the continuous and discrete solutions of adsorption is important. The difficulty of understanding lies in the self-similar fractal nature of diffusion and its probability density function. From a finite-difference point of view, the broadening of the ensemble diffusion profile is a combination of the broadening of a smaller fraction of the earlier profiles (Fig. 2). This creates a question on directly using the combined ensemble curves [Eq. (2)] to calculate the adsorption probability of a molecule from the bulk to the surface. Non-Gaussian diffusion is often observed in a confined space or near a surface further complicated the problem.^7,16^ The adsorption of the molecules is greatly affected by the reabsorption of the reflected molecules.^8^ It is becoming important for us to re-evaluate the theory of adsorption under ideal conditions to reduce the difficulty in building or understanding more complicated models.

In this report, we are comparing this theory with the results of Monte Carlo simulations to revisit the correlation between the continuous theoretical models with the discrete simulation results.^25^ An additional task of the comparison is to gain insights into a gap between the theory and experiments.^24,26–32^ The Ward–Tordai (WT) equation is widely used to measure the effective diffusion constant from the experimental data.^14,32^ However, the effective diffusion constant is often several orders of magnitude different from the value predicted by the Stoke–Einstein equation.^14,32^ These complexities have limited the applications of the Ward–Tordai equation [Eq. (5)] in many fields, such as chemistry, biochemistry, biophysics, biotechnologies, and chemical engineering.^29,30^ For example, it is critical to predict how long it takes for a drug molecule to be adsorbed on the surface of a cell and how long it takes to diffuse to the target binding site inside a cell. It is also important to predict how long we should wait for a typical biosensing platform, such as a glucose sensor and a surface plasmon resonance sensor. Predicting the diffusion of a molecule to the surface is also essential to calculate the corrosion rate of a pipe and the reaction rates in a heterogeneous catalytic system.

## RESULTS AND DISCUSSION

**FIG. 3.**
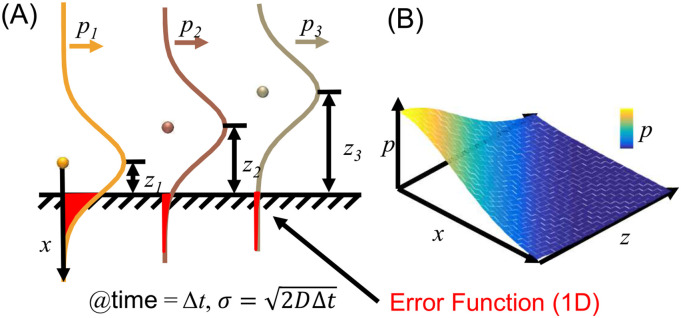
(a) A simple method in obtaining the adsorption rate is by integrating the overall probability function and ignoring the fractal nature of the diffusion. Snapshot of particles (dots) showing at their origins and scheme of their 1D diffusing PDFs (Gaussian) over space at time Δ*t* (colored curves). The probability of each particle hitting an imaginary interface perpendicular to its diffusing direction is shown in the red-colored error functions ignoring its fractal nature and the mirror effect. (b) A scheme to integrate all the error functions.

In a typical measurement, we often monitor the accumulated molecule adsorbed on the surface with a time resolution *τ* as the integration time of each measurement step, e.g., a frame of a movie. Assuming the locations of the molecules are known at the beginning of each frame, whose diffusion probabilities will create broadening profiles described by Eq. (2) by replacing the *t* with ∆*t* = *τ* (Fig. 3). If we integrate the error functions of all these profiles in the bulk solution (Fig. 3 and supplementary material), this will represent the adsorption simulated using the same discrete-time resolution,

$$\Gamma \left(in\;\Delta t\right)=Ac_{b}\sqrt{\frac{D\Delta t}{\pi }}.$$ (7)

Comparing Eq. (5) with Eq. (7), we can see that the discrete solution has a two-fold reduction in predicting the adsorption by ignoring the fractal diffusion that happens within ∆*t*. When we zoom in the time of the frame at when a molecule hits the surface, the probability function collapses into a binary state: it either adsorbs, which truncates the average PDF, or it reflects, which increase the probability of the adsorption in the next moment by the same amount as the truncated error function. We can call this doubling a “mirror effect,” which analogies to Zeno’s paradoxes. As such, the overall probability of adsorption is twice the sum of the error functions shown in Fig. 3,

$$\Gamma \left(in\;\Delta t\right)=2Ac_{b}\sqrt{\frac{D\Delta t}{\pi }}.$$ (8)

Equation (5) assumes that the adsorption continues from frame to frame and the concentration gradient near the surface continuously evolves (the concentration decreases over time since the molecules are absorbed by the surface), while Eq. (8) assumes that the concentration gradient evolves during the single frame and then recovers to the original at the beginning of the next frame. Equation (8) can be used to predict the number of collisions of the molecules to a non-stick surface with care being taken for choosing the length of ∆*t*. The average rate of solute colliding the wall measured in ∆*t* is

$${\left\langle r\right\rangle }_{\left(meausred\;in\;\Delta t\right)}=2Ac_{b}\sqrt{\frac{D}{\pi \Delta t}}.$$ (9)

Equation (9) is confusing that the rate of collision depends on the observation time. As such, we run Monte Carlo simulations to reproduce the result of Eq. (8) on a reflective surface and Eq. (5) on an adsorptive surface.

**FIG. 4.**
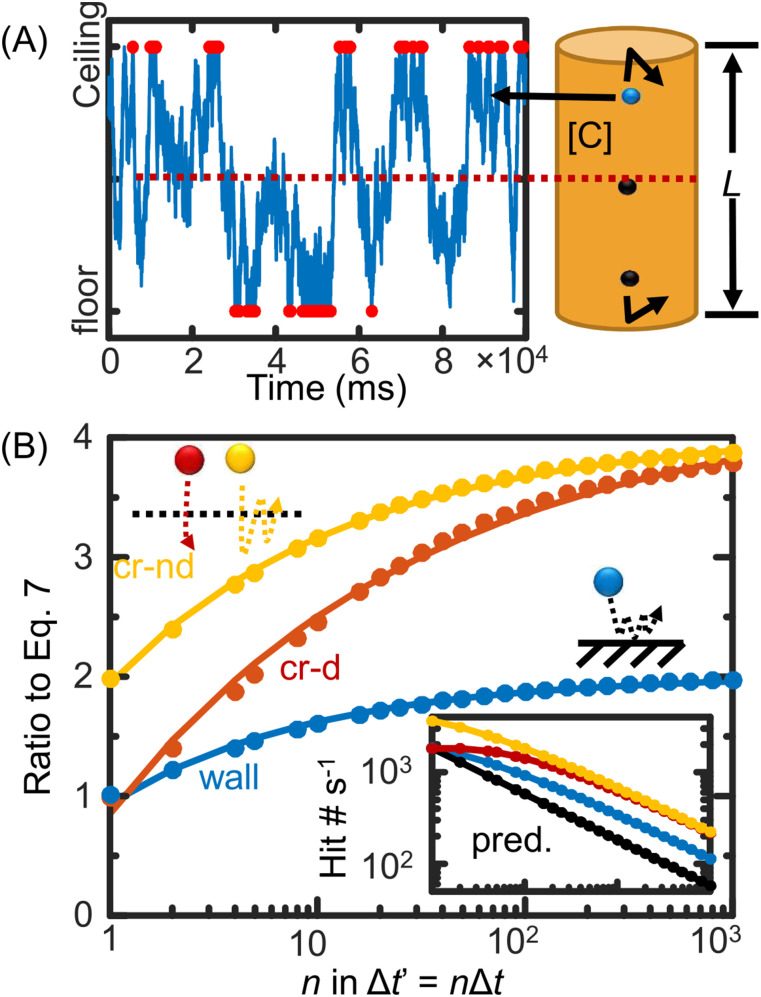
Metropolis Monte Carlo simulation of 1D diffusive molecules in [(a) and (b)] a cylindrical volume that bounces the molecules at both ends. (a) The trajectory of a randomly chosen molecule over time. (b) Averaged number of molecules collide with the walls (wall, blue), passing the interface from one direction (cr-d, red), or either direction (cr-nd, yellow) within the binned observation time. Multiple collisions from the same molecule in one cycle are counted once. The number in the y axis is normalized to the predicted values using Eq. (7).

Figure 4 shows Monte Carlo simulations of molecules moving in a 1D space that bounced/reflected from both ends. Equation (7) is reproduced from the Monte Carlo simulation using a single PDF. Equation (8) is reproduced by binning >1000 simulation steps to mimic the finer fractal diffusion by combining the PDFs with sub-frame resolution. The time-dependent collision rate is confirmed to cause by the repetitive collision of the same molecule in a measuring cycle if observed at a finer time resolution. A real mirror effect, doubling crossing probability, is also observed at an imaginary interface in the bulk. See the supplementary material for a discussion of Fig. 4 results.

**FIG. 5.**
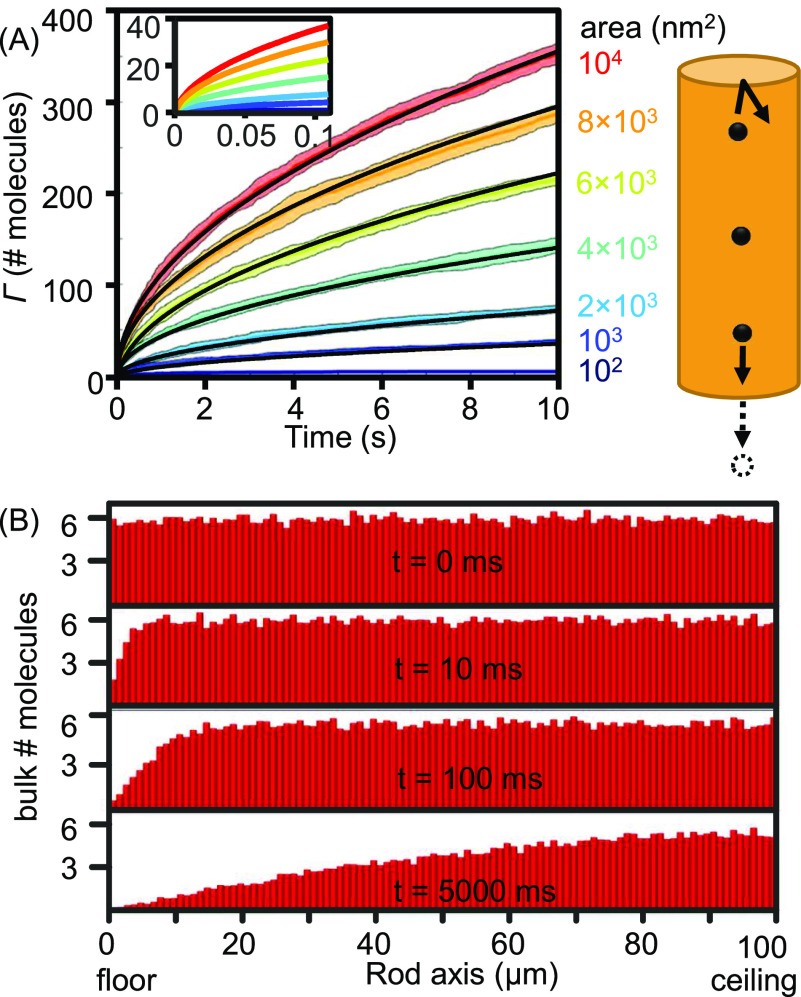
Metropolis Monte Carlo simulation of 1D diffusive molecules in a cylindrical volume that bounces on the ceiling but “absorbs” on the floor. (a) The number of molecules adsorbed on the floor over time overlaid with the predicted values using Eq. (5). (b) Molecular concentration gradient evolving in the rod volume over time.

Figure 5 shows Monte Carlo simulations of molecules moving in a 1D space that bounced/reflected from one end and adsorbed on the other. Because one adsorption per simulated step has been satisfied in such a simulation, no binning is required to reproduce the adsorption kinetics predicted by Eq. (5). We can also visualize the evolution of the concentration gradient near the adsorptive surface and the bulk over time. This gradient is originally rationalized in developing the Langmuir–Schaefer and Ward–Tordai equations that the adsorption rate drops over time.

Comparing the two simulations in Figs. 4 and 5, a confusing question raises: During a discrete measurement, what ∆*t* one should choose to calculate the “initial” rate of the adsorption? The initial rate is commonly used in the kinetic analysis in the literature. During the first discrete measuring cycle, time 0 − Δ*t*, do we consider an evolving sub-surface-bulk concentration gradient or do we consider a uniform concentration across the bulk as time 0?

**FIG. 6.**
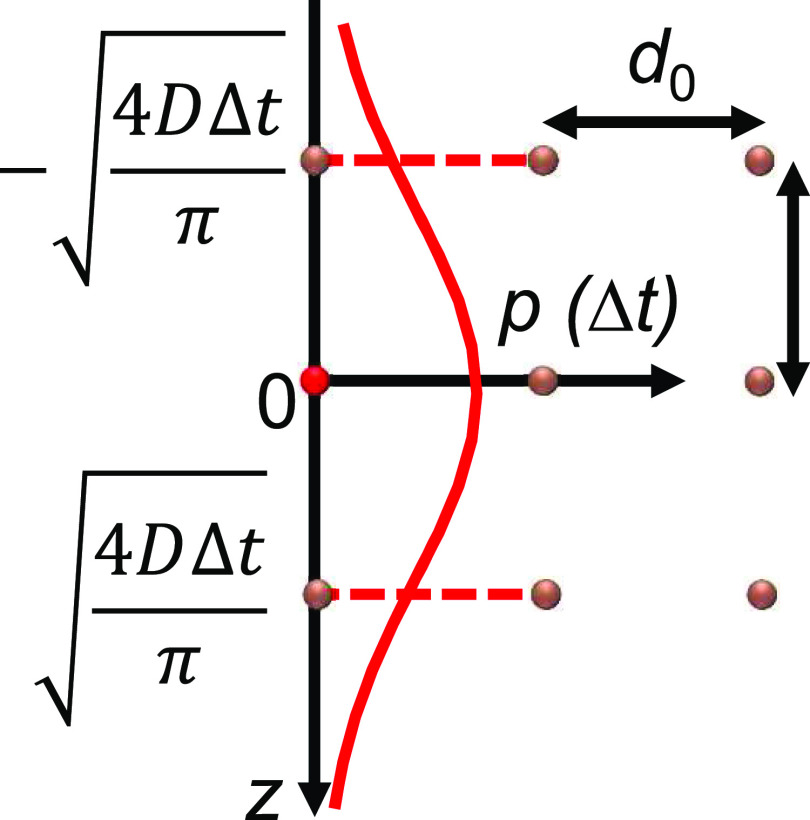
Scheme of finding characteristic Δ*t* to calculate the adsorption frequency when the sub-surface concentration is the same as the bulk concentration, i.e., the short-time limit right after a fresh surface is exposed to the solution.

We can do a mind experiment with molecules aligned perfectly in space shown in Fig. 6. To maintain the same molecular distribution within the time Δ*t*, the average location of the molecule should be the same as the distance between two molecules, i.e., the net effect is just switch positions during this time,

$$d_{0}=\frac{1}{\sqrt[{3}]{c_{b}}}=\frac{\int_{0}^{\infty }ze^{\frac{-z^{2}}{4D\Delta t}}dz}{\int_{0}^{\infty }e^{\frac{-z^{2}}{4D\Delta t}}dz}=\sqrt{\frac{4D\Delta t}{\pi }},$$ (10)

where *d*_0_ and *z* are shown in Fig. 6. Thus, the characteristic integration time ∆*t*_c_ to calculate the average adsorption rate with no sub-surface concentration gradient is

$$\Delta t_{c}=\frac{\pi }{4D{C_{b}}^{2/3}}.$$ (11)

Thus, the average initial adsorption rate can be calculated by Eq. (9),

$$\left\langle r\right\rangle =2Ac_{b}\sqrt{\frac{D}{\pi \Delta t_{c}}}=4\pi^{-1}A{c_{b}}^{4/3}D.$$ (12)

This equation has the correct unit s^−1^ for the dimensional analysis and is consistent with another calculation, assuming the molecular exchanging time ∆*t*_c_ is the average adsorption time for the characteristic surface area *d*_0_^2^. That is,

$$\left\langle r\right\rangle =\frac{A}{d_{0}^{2}}\frac{1}{\Delta t_{c}}=4\pi^{-1}A{c_{b}}^{4/3}D.$$ (13)

In short, ∆*t*_c_ is the characteristic diffusion-limit time for the adsorption. Shorter than this time, the high average hitting rate and the low total number of adsorptions predicted by Eqs. (8) and (9) are due to the isolated but repetitive collision of the same molecules on the surface. Longer than this time, the same molecule might have diffused away beyond the first nearby layer and diffused back to the surface, which should have been counted as “different” molecules if the sub-surface concentration has been maintained, i.e., Eq. (9) has lower estimated the collision frequency longer than this time. Thus, at the very beginning of the adsorption, ∆*t*_c_ is the time between the molecules that have just collided with the surface and diffused away to the first nearest neighbor layer.

Equation (13) derived from the single-molecule approach is consistent with the results from the ensemble method. Equation (13) predicts values that are consistent with the diffusion-limited steady-state flux of Pt nanoparticles on an ultramicroelectrode (UME).^33^ When diameter of UME is *r* = 10 *μ*m, *c*_b_ = 25 pM, and D = 1 × 10^−12^ m^2^ s^−1^, the critical adsorption rate is calculated as 0.37 s^−1^. This rate is consistent with the calculated value as 0.4 s^−1^ using the semiempirical steady-state flux equation.^33,34^

The 4/3-order dependence of rate on concentration is very weird, and I initially also think it is wrongly derived. For the adsorption, Eq. (9) already shows that it should be the first-order dependent on the concentrations; thus, the 4/3-order dependence in Eq. (13) is only for the initial rate at a short period when the symmetry is just broken by introducing the plane in the solution. It is unlikely to observe this dependence in a real adsorption experiment when a concentration gradient will develop. It is a useful initial assessment of the order of rate that is independent of the observation integration time, especially for experiments on a system with a small absorption area surrounded by a much larger inactive area and/or with a fast flow rate.^4,6,7,9,23,25,35–38^ However in the bulk solution, the 4/3-order dependence of the diluted concentration on the collision order is surprising. In the physical chemistry textbooks, first-order dependence for high concentration gas reaction is supported by the collision theory, where collision frequency is a first-order function of concentration. This fraction order is consistent with previous simulations on the fractal nature of reaction kinetics that obtains abnormal reaction orders for simple reactions.^39^

In the literature, the Ward–Tordai equation often gives several orders of magnitude differences between the measured effective diffusion constant and that predicted by the Stokes–Einstein equation. Thus, it is interesting to simulate the adsorption of molecules on a surface using the typical Langmuir surface adsorption model. We are simulating two systems, self-assembled monolayers (SAMs) and binding of molecules to a patterned surface. SAMs are used in many fields for surface functionalization and the latter is often used in biosensing systems, where both need a simple equation to predict the adsorption kinetics.

**FIG. 7.**
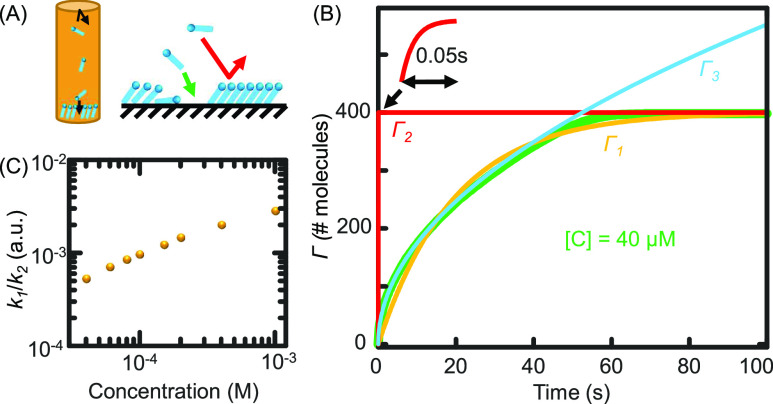
Monte Carlo (Gillespie algorithm) simulation of the formation of SAMs. (a) Simulation scheme of SAMs. (b) A selected simulating curve (green) overlapped with three fitting and calculated curves explained in the main text. (c) The ratio between the fitted rate constant vs the predicted rate constant with an ideal stirring for different bulk concentrations was simulated.

The Langmuir adsorption of SAM without energy barriers is simulated using the Gillespie algorithm, a computer-based Monte Carlo simulation method.^40,41^ Specifically, the bulk solute molecules with simulated 1D diffusion adsorb on empty sites and are rejected on occupied sites on the surface [Fig. 7(a)]. A Ward–Tordai (WT) adsorption curve is observed with both the adsorption and back-diffusion simulated [Fig. 7(b), green curve]. These simulations are empirically consistent with typical experiments on SAMs, mostly saturate in a few minutes under similar conditions.^42^ The Ward–Tordai curve can be approximated to an exponential decay function with deviations at the early and late part of the curve,

$$\Gamma_{1}=\frac{A}{a}\left(1-e^{-k_{1}t}\right),$$ (14)

where *A* is the total surface area simulated, *a* is the average size of a binding site, *t* is time, and *k* is the effective rate constant on the surface. The effective rate constant can be pulled out from fitting and labeled as *k*_1_, and the fitted curve is labeled *Γ*_1_ [Fig. 7(b), orange curve].

If an ideal stirring has been introduced that maintains the bulk concentration with no gradient at the front edge of the surface, an exponential decay curve *Γ*_2_ with the same model as *Γ*_1_ but with much faster rate constant *k*_2_ than *k*_1_ would have been observed [Fig. 7(b), red curve] with the rate constant,

$$k_{2}=4\pi^{-1}a{c_{b}}^{4/3}D.$$ (15)

Surprisingly, Eq. (5) is often considered short-time limited in the literature and overlaps with the simulated data most of the time until ∼80% of saturation [Figs. 7(b), *Γ*_3_]. Equation (5) has not considered the shrinking active binding area of the SAMs (due to increasing adsorption coverage) and the slowing-down evolution speed of the concentration gradient (due to the back-diffusion) near the surface over time. Coincidentally, these two effects cancel with each other during most of the adsorption time.

The difference larger than three orders of magnitude between *k*_1_ and *k*_2_ [Fig. 7(c)] explains the over six orders of magnitude variations of the effective diffusion constant calculated from the Ward–Tordai equation on experimental data, which is still confusing nowadays in the literature.^14,32^ We can speculate an answer from the simulation results that because convection, flow, and stirring naturally occur under typical experimental conditions, greatly changing the formation of the concentration gradient near the surface.

**FIG. 8.**
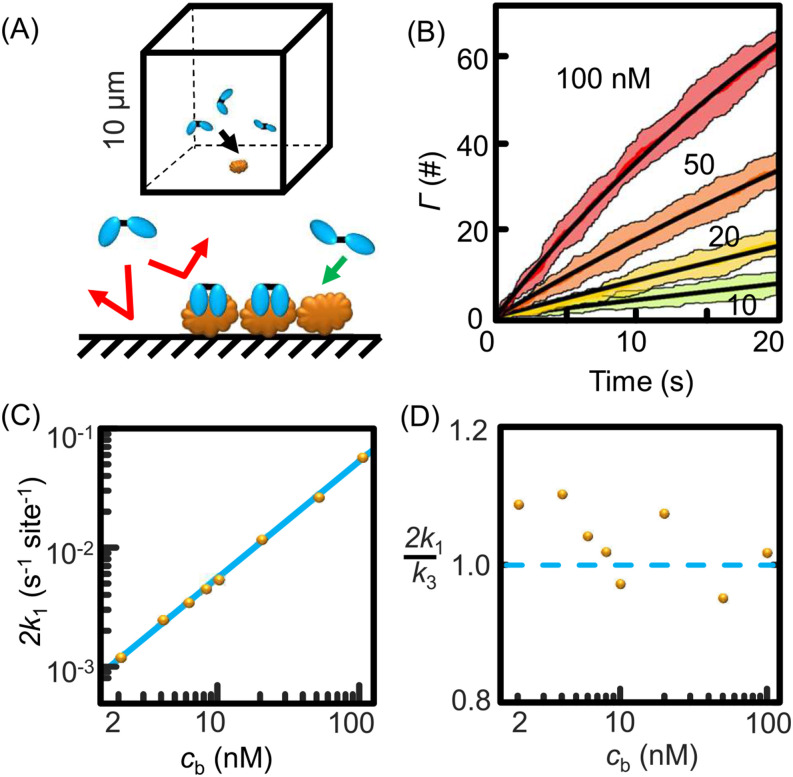
Monte Carlo (Gillespie algorithm) simulation of biosensing on a patterned surface. (a) Scheme for the biosensing platform simulated a 10-nm radius circle with 150 binding sites. Immobilized binding sites are brown and diffusive probes are blue. (b) Adsorption curves with different bulk concentrations. The black curve is the fitting overlaid on the colored belt representing the standard deviation of ten simulations. (c) Fitted rate constants times 2 (yellow dots) follow a linear correlation with the concentration. Rate constants *k*_3_ are calculated from Eq. (16) and overlaid as the blue line. (d) The ratio between two times the fitted rate constants (yellow dots) and the predicted rate constants over different bulk concentrations simulated (*k*_3_ are normalized to 1 shown by the dashed blue line). Derivation of the adsorption equation from 1D to 3D is detailed in the supplementary material.

We can simulate the initial slope predicted by Eq. (9) with a much higher consistency with experimental results than SAMs for a special case. We simulate a typical biosensing system on a patterned surface and/or under significant flow (Fig. 8) when the sub-surface concentration of the solution does not change much throughout adsorption. This is simulated with a 3D diffusion model (see the supplementary material for detailed derivation and simulation conditions). Figure 8(a) shows the simulation scheme with a 10-nm-radius adsorption spot (with 150 binding sites, each occupies ∼2 nm^2^) on the wall of a 1000-*μ*m^3^ cubic volume. The rate constants of Fig. 8(b) are fitted with Eq. (14) and shown in Fig. 8(c).

By replacing the ensemble area with the single-molecule binding area, Eq. (9) predicts rate constants,

$$k_{3}=2ac_{b}\sqrt{\frac{D}{\pi \Delta t}},$$ (16)

where *a* (m^2^) is the area of each binding site, *c*_b_ (# m^−3^) is the bulk concentration, *D* (m^2^ s^−1^) is diffusion constant, and ∆*t* is the step time 0.001 s.

Twice the simulated binding constant *k*_1_ [Eq. (14)] is compared to the theoretical values *k*_3_ [Eq. (16)] [Fig. 8(d)]. Twice is chosen to correct the fractal diffusion being ignored during the simulation when the majority of the surface is reflective. We can conclude from the consistency between the two values that Eq. (9) holds for such simulations [Fig. 8(d)], suggesting that this equation is applicable in biosensing and drug delivery fields. These simulations are consistent with experimental results reported in the literature where single-molecule adsorption of dye molecules on immobilized DNA is measured^43^ or protein-surface adsorption within an order of magnitude.^24^

These simulations also suggest that the reverse argument is true, that is, for binding of diffusive solute molecules to isolated targets on a surface, Eq. (5) can be used to measure the diffusion constant of the solute, overcoming the several orders of magnitude variation observed in the measurements of SAMs.

## CONCLUSIONS

In summary, the surface adsorption kinetics represented by the Langmuir–Schaefer equation and the Ward–Tordai equation has been reproduced using Monte Carlo simulations. The fractal nature of diffusion is examined and simulated. A unique conclusion suggested by the single-molecule approach is that there is a characteristic integration time ∆*t*_c_ for the equations, which has never been a consideration for the ensemble approaches, a missing piece of the Ward–Tordai equation. That is, the characteristic time distinguishes the overestimated multiple collisions from the same molecule shorter than this time and the lower estimation of the collisions longer than this time. We can draw some interesting specific conclusions from the Monte Carlo simulations. For example, when we measure the collision events of probe molecules in the bulk solution to a small target area on a flat surface, the frequency of seeing such events is dependent on the measuring time resolution and how the surface reacts with the probes. With the results obtained from the simulations, we may start to use the simple continuous and discrete equations carefully in various fields, such as calculating the collision frequency of molecules in a diluted gas or liquid solution, membrane penetration, self-assembly, and biosensing. Wide use of these equations in these fields has not yet been seen in the literature. It may also find applications in finer simulations such as molecular dynamics simulations to skip non-interested mass transportation steps among the solvent. More careful simulations and experiments should be carried out in the future to further test the limit of these equations. Nevertheless, I hope you agree that the single-molecule approach pictured in this report is easier to understand than the ensemble equations of diffusion.

## METHODS

The Monte Carlo simulation is carried out on a laptop equipped with an Intel i7 CPU (2.2 GHz) and 16 Gb of memory. A basic version of MATLAB 2014b is used for all simulations. A single central processing unit (CPU) is used for all simulations. A previously coded exponential regression fitting algorithm *fitNguess*^44^ (Github) is used to fit the curves. The two major random functions to generate the step motion of each molecule are from MATLAB, *rand*() creating evenly distributed random numbers, and *randn*() generating Gaussian distributed random numbers. Detail parameters and settings for the simulations are listed in the supporting information. The single-molecule diffusion of the solute molecules in confined semi-infinite volumes and their collisions to the walls are simulated. Inter-molecular collisions are not simulated. Typical computer time used for the simulations is from a few seconds to a few hours. See the last sections of the supplementary material for two example source codes in MATLAB.

## SUPPLEMENTARY MATERIAL

See the supplementary material for simulation details, derivations, and example MATLAB source codes for 1D and 3D simulations. Other source codes and data are available upon request.

## Data Availability

MATLAB source codes have been included in the supplementary material. Additional codes and data that support the findings of this study are available from the corresponding author upon reasonable request.
